# The effect of dichotomization of skewed adjustment covariates in the analysis of clinical trials

**DOI:** 10.1186/s12874-023-01878-9

**Published:** 2023-03-13

**Authors:** Alan Herschtal

**Affiliations:** School of Public Health and Preventive Medicine, Monash University, 553 St Kilda Rd, Melbourne, 3004 Australia

**Keywords:** Clinical trials, Covariates, Adjustment, Stratification, Linear regression, Skewness

## Abstract

Baseline imbalance in covariates associated with the primary outcome in clinical trials leads to bias in the reporting of results. Standard practice is to mitigate that bias by stratifying by those covariates in the randomization. Additionally, for continuously valued outcome variables, precision of estimates can be (and should be) improved by controlling for those covariates in analysis. Continuously valued covariates are commonly thresholded for the purpose of performing stratified randomization, with participants being allocated to arms such that balance between arms is achieved within each stratum. Often the thresholding consists of a simple dichotomization. For simplicity, it is also common practice to dichotomize the covariate when controlling for it at the analysis stage. This latter dichotomization is unnecessary, and has been shown in the literature to result in a loss of precision when compared with controlling for the covariate in its raw, continuous form. Analytic approaches to quantifying the magnitude of the loss of precision are generally confined to the most convenient case of a normally distributed covariate. This work generalises earlier findings, examining the effect on treatment effect estimation of dichotomizing skew-normal covariates, which are characteristic of a far wider range of real-world scenarios than their normal equivalents.

**Supplementary Information:**

The online version contains supplementary material available at 10.1186/s12874-023-01878-9.

## Introduction

Two-arm randomized clinical trials with a continuous valued outcome may be analysed using a linear regression model to test for association between the dichotomous intervention (independent variable), and the outcome (dependent variable). As with all tests for association between an intervention and an outcome, it is important to adjust for any baseline covariates believed a priori to be associated with the outcome [1–6]. This protects against bias due to baseline imbalance and increases the precision of treatment effect estimates.

When the baseline covariate to be controlled for is either categorical or ordinal, a common approach for this adjustment consists of two steps. Firstly, each level of the baseline covariate is regarded as a separate stratum and the randomisation is stratified such that the desired study-wide allocation ratio is honoured in each stratum individually. This stratified randomization approach pre-empts any incidental imbalance in the covariate between arms which may arise in simple randomization due to sampling variability.

Then, at the analysis stage, the baseline covariate is controlled for by including it as an additional independent variable in the model. This partitions the variance between the baseline covariate and the intervention and thus yields a more precise estimate of the treatment effect. Failing to adjust for stratification variables in analysis leads to models which overestimate standard error, and thus overestimate confidence interval width, underestimate type 1 error, and reduce power [1, 5].

The procedure above is easily implemented for categorically valued baseline covariates such as gender or ethnicity, or ordinal ones such as disease stage. However, when the baseline covariate to be adjusted for is continuously valued, such as patient age or BMI, no naturally occurring strata exist, and more variability exists in approach [7]. Creating artificial strata by thresholding the baseline covariate at pre-defined bin boundaries is attractively simple, as it allows the stratified randomization to proceed in the same way as for the categorical or ordinal covariate above. Although the decision as to how many bins to threshold into and what the bin boundaries should be introduces a certain arbitrariness into the adjustment, it is nonetheless widespread practice, and often a simple dichotomization at a somewhat arbitrarily chosen value close to the median is deployed. Thus, for example, prior to inclusion as covariates in a model, age may be dichotomized as < 55 vs. $\ge$ 55 years, BMI as < 30 vs. $\ge$ 30 kg/m^2^, and continuously valued gene-based risk scores may be summarized as ‘high’ vs. ‘low’, based on a pre-determined threshold.

Unfortunately, when it comes to analysis, oftentimes the stratification variables are included in the model using the same dichotomization that was used in the stratification. It is well documented that this leads to substantial additional imprecision in treatment effect estimates, and is subject to all the same drawbacks as omitting the stratification variable from analysis altogether, only to a lesser extent [8–10].

Analytic approaches to understanding and quantifying the deleterious effect of covariate dichotomization in the literature have focussed, for simplicity and convenience, on the case where the covariate of concern is normally distributed [4]. However, non-normally distributed covariates arise frequently in the analysis of medical data in particular, and are a subject of increasing interest in clinical trials. It is well documented that anthropomorphic measures such as BMI [11, 12] and weight [13], lipid measurements such as triglycerides [14], biomarker measurements, and commonly used measures in medical domains as diverse as opfthalmology [15] and cardiology [16] all display substantial right skew.

Log-transformation, the most commonly used method of normalizing right skewed data, is inflexible and in many cases will either over- or under-correct for the skewness. More flexible normalization methods such as the Box-Cox transformation [17] have been used with some success in normalizing skewed anthropomorphic data [18] but come at the cost of potentially undermining the assumed linear relationship between the covariate and the outcome variable. It is thus of considerable interest to extend findings on the effect of covariate dichotomization from the case of normally distributed covariates to that of skew-normal ones.

## Method

Consider a test for association between a dichotomous indicator variable $z$ representing treatment (intervention vs. standard) and a continuously valued outcome variable $y$, controlling for a skew-normal (SN) distributed covariate $x$ purportedly associated with $y$. Assuming a linear relationship between $x$ and $y$, the following model may be considered:

1 $$y=\alpha +\gamma z+\beta x+\varepsilon$$

where $\varepsilon \sim N\left(0,{\sigma }_{\varepsilon }\right)$ and $x \sim SN\left(\varphi ,\omega ,\lambda \right)$. $\varphi$ and $\omega$ are the location and scale parameters of $x$ respectively. $\lambda$ controls the skewness. If $\lambda =0$ the normal distribution is retrieved. The variance of $x$is given by [19, 20].

2 $${\sigma }_{x}^{2}={\upomega }^{2}\left(1-\frac{2{\lambda }^{2}}{\pi \left(1+{\lambda }^{2}\right)}\right)$$

The test for a treatment effect is formulated as a hypothesis test with null hypothesis of $\gamma =0$ against a 1-sided alternative ($\gamma >0$ or $\gamma <0$), or a 2-sided alternative ($\gamma \ne 0)$. The precision of the estimator of $\gamma$, $\widehat{\gamma }$, affects the power of the hypothesis test, the confidence interval width and the p-value. It is thus of interest to assess the effect of dichotomization of the covariate $x$ on the precision of $\widehat{\gamma }.$ To this end, we compare the following three models:

i) the full model as presented in Eq. 1;
ii) a restricted model, in which the covariate $x$ is omitted;
iii) a ‘partially restricted’ model, in which $x$ is dichotomized before inclusion in the model.

The full model takes advantage of all the information available in $x$, whereas the restricted model does not use $x$ at all. The partially restricted model resides somewhere between these extremes. Measuring the precision of $\widehat{\gamma }$ under the partially restricted model relative to the full and restricted models tells us just how much information is lost by dichotomization of a SN covariate when estimating a treatment effect.

We denote the total sample size by $n$, and consider a 1:1 allocation ratio, ($n/2$ participants per arm).

i) **Full model**

From analysis of variance theory [21] we have that for the linear model in Eq. 1, the variance of $\widehat{\gamma }$, $V\left(\widehat{\gamma }\right)$, may be expressed as

$$V\left(\widehat{\gamma };m{}_{\mathrm{f}}\right)={\sigma }_{\varepsilon }^{2}/{S}_{zz}$$

where $m{}_{\mathrm{f}}$ represents the full model and ${S}_{zz}$ is the sum of squared errors for $z$:

$${S}_{zz}= \sum_{i=1}^{n}{\left(z{}_{i}-\overline{z }\right)}^{2}$$

Encoding the arm indicator $z{}_{i}$ as 0 (standard care) or 1 (intervention), for 1:1 randomization, $\overline{z }=\frac{1}{2}$, ${\left(z{}_{i}-\overline{z }\right)}^{2}=\frac{1}{4} \forall i$, ${S}_{zz}=\frac{n}{4}$ and thus

$$V\left(\widehat{\gamma };m{}_{\mathrm{f}}\right)={4\sigma }_{\varepsilon }^{2}/n$$

ii) **Restricted model**

Because we are considering a randomized study, $x$ and $z$ can be expected to be independent. In this case, if the covariate $x$ is omitted from the model altogether, the mean component of the $\beta x$ term will be absorbed into the intercept $\alpha$ and the variance component of the $\beta x$ term, ${\beta }^{2}{\sigma }_{x}^{2}$, will be absorbed into the error term, $\varepsilon$, whose standard error under the restricted model will be referred to as ${{\sigma }_{\upvarepsilon }}^{^{\prime}}$.

$${{\sigma }_{\upvarepsilon }}^{^{\prime}} = \sqrt{{\sigma }_{\varepsilon }^{2}+{\beta }^{2}{\sigma }_{x}^{2}}$$

Using $m{}_{\mathrm{r}}$ to denote the restricted model, and using the expression for ${\sigma }_{x}^{2}$ in Eq. 2,

$$V\left(\widehat{\gamma };m{}_{\mathrm{r}}\right)=4\left({\sigma }_{\varepsilon }^{2}+{\beta }^{2}{\omega }^{2}\left(1-\frac{2{\lambda }^{2}}{\pi \left(1+{\lambda }^{2}\right)}\right)\right)/n$$

iii) **Partially restricted model**

We now consider the effect of dichotomizing SN distributed covariate $x$ prior to including it in the model.

The SN distribution may be expressed as [22].

$$f\left(x;\varphi ,\omega ,\lambda \right)=\frac{2}{\omega }\phi \left(\frac{x - \varphi }{\omega }\right)\Phi \left(\lambda \frac{x -\varphi }{\omega }\right)$$

$\phi \left(.\right)$ represents the standard normal distribution and $\Phi \left(.\right)$ is its cumulative distribution. When $\lambda =0$ the normal distribution is recovered.

For notational convenience, without loss of generality, we temporarily restrict analysis to the special case of $\varphi =0$ and $\omega =1$. The expected value of a doubly truncated standard SN random variable can then be expressed in terms of $\lambda$ and the lower and upper standardized truncation points, $\alpha$ and $\beta$ respectively [21, 23, 24].

3 $$E\left(x;\lambda | \alpha <x<\beta \right)= - \frac{f\left(\beta ;\lambda \right)-f\left(\alpha ;\lambda \right)}{F\left(\beta ;\lambda \right)-F\left(\alpha ;\lambda \right)}+{\omega }^{2}\sqrt{\frac{2{\lambda }^{2}}{\pi \left(1+{\lambda }^{2}\right)}}\frac{\Phi \left(\beta \sqrt{1+{\lambda }^{2}}\right)-\Phi \left(\alpha \sqrt{1+{\lambda }^{2}}\right)}{F\left(\beta ;\lambda \right)-F\left(\alpha ;\lambda \right)}$$

$f\left(.;\lambda \right)$ and $F\left(.;\lambda \right)$ are the distribution and cumulative distribution functions respectively of the standard SN distribution with skewness parameter $\lambda$, and $\Phi \left(.\right)$ is the cumulative distribution function of the standard normal.

Consider that $x$ may be partitioned into two component random variables. The first, denoted by ${x}_{d}$, represents the dichotomized $x$. For $x$ below the dichotomization threshold $u$, ${x}_{d}$ is set to ${u}_{-}$, the mean of all values of $x$ below $u$. For $x$ above $u$, ${x}_{d}$ is set to ${u}_{+}$, the mean of all values of $x$ above $u$. The second random variable, denoted by ${x}_{r}$, is the “residual” of $x$ around ${x}_{d}$, ${x}_{r} = x - {x}_{d}$. Setting the lower and upper values of ${x}_{d}$ in this way achieves independence of ${x}_{d}$ and ${x}_{r}$, such that $V\left(x\right)=V\left({x}_{d}\right)+V\left({x}_{r}\right)$. Proof of this can be found in Additional file 1: Appendix 2.

To calculate the variance of ${x}_{d},$ we require the mean of $x$ above and below the dichotomization point (singly truncated means), as well as the overall (untruncated) mean. These are arrived at by setting the boundary points $\alpha$ and $\beta$ in Eq. 3 to $\alpha =-\infty$ and $\beta =u$ or to $\alpha =u$ and $\beta =\infty$ for the singly truncated means, and to $\alpha =-\infty$ and $\beta =\infty$ for the untruncated mean. By definition, $f\left(-\infty ;\lambda \right)=f\left(\infty ;\lambda \right)=0$, $F\left(-\infty ;\lambda \right)=0, F\left(\infty ;\lambda \right)=1$, and $\Phi \left(-\infty \right)=0,\Phi \left(\infty \right)=1$. For dichotomization threshold $u$, we have

$$E\left(x|-\infty <x<u\right)= - \frac{f\left(u;\lambda \right)}{F\left(u;\lambda \right)}+\sqrt{\frac{2{\lambda }^{2}}{\pi \left(1+{\lambda }^{2}\right)}}\frac{\Phi \left(u\sqrt{1+{\lambda }^{2}}\right)}{F\left(u;\lambda \right)}$$

and

$$E\left(x| u<x<\infty \right)= \frac{f\left(u;\lambda \right)}{1-F\left(u;\lambda \right)}+\sqrt{\frac{2{\lambda }^{2}}{\pi \left(1+{\lambda }^{2}\right)}}\frac{1-\Phi \left(u\sqrt{1+{\lambda }^{2}}\right)}{1-F\left(u;\lambda \right)}$$

The undichotomized mean is also easily derived as

$$E\left(x\right)= \sqrt{\frac{2{\lambda }^{2}}{\pi \left(1+{\lambda }^{2}\right)}}$$

We represent the percentile at which dichotomization occurs as $\tau$, $0<\tau <1$, i.e. $\tau =F\left(u;\lambda \right)$.

Scaling by $\omega$ to retrieve the more general case of $x \sim SN\left(\varphi ,\omega ,\lambda \right)$, the variance of ${x}_{d}$ may be calculated using the above relationships for the truncated means and the identities $Var[X]=E\left[{X}^{2}\right]-{E\left[X\right]}^{2}$ and $\mathrm{Var}\left(X\right)=\frac{1}{n}{\sum }_{i=1}^{n}{\left({x}_{i}-\mu \right)}^{2}$.

$$V\left({x}_{d}\right)={\frac{{\omega }^{2}}{\tau \left(1-\tau \right)}\left(f\left({F}^{-1}\left(\tau \right)\right)+\sqrt{\frac{2{\lambda }^{2}}{\pi \left(1+{\lambda }^{2}\right)}}\left(\tau -\Phi \left({F}^{-1}\left(\tau \right)\sqrt{1+{\lambda }^{2}}\right)\right)\right)}^{2}$$

Since $V\left(x\right)=V\left({x}_{r}\right)+V\left({x}_{d}\right)$, we have partitioned the variance attributable to $x$ into a component attributable to ${x}_{d}$ and another attributable to ${x}_{r}$. We may now calculate the variance of $\widehat{\gamma }$ under the partially restricted model as follows.

After partitioning $x$ into components ${x}_{d}$ and ${x}_{r}$, the model may be expressed as:

$$y=\alpha +\gamma z+\beta {x}_{r}+\beta {x}_{d}+\varepsilon$$

Arguing analogously as for the restricted model, since ${x}_{r}$ is independent of $z$, if the covariate ${x}_{r}$ is omitted from the model, then the mean component of the $\beta {x}_{r}$ term will be absorbed into $\alpha$ and the variance component of the $\beta {x}_{r}$ term, ${\beta }^{2}\left(V\left(x\right)-V\left({x}_{d}\right)\right)$, will be absorbed into the error term, $\varepsilon$, whose standard deviation will now be referred to as ${\sigma }_{\upvarepsilon }\mathrm{^{\prime}}\mathrm{^{\prime}}$.

$${\sigma }_{\upvarepsilon }\mathrm{^{\prime}}\mathrm{^{\prime}} = \sqrt{{\sigma }_{\upvarepsilon }^{2}+{\beta }^{2}\left(V\left(x\right)-V\left({x}_{d}\right)\right)}$$

Denoting the partially restricted model by ${m}_{\mathrm{p}}$,

$$V\left( \widehat{\gamma };{m}_{\mathrm{p}}\right)=4\left({\sigma }_{\upvarepsilon }^{2}+{\beta }^{2}\left(V\left(x\right)-V\left({x}_{d}\right)\right)\right)/n$$

The reduction in variance associated with the estimator for $\widehat{\gamma }$ when going from the restricted model (covariate $x$ omitted altogether) to the full model (covariate $x$ included in raw form) can be derived by subtraction.

$$V\left(\widehat{\gamma };{m}_{\mathrm{r}}\right)-V\left(\widehat{\gamma };{m}_{\mathrm{f}}\right)=4{\beta }^{2}{\sigma }_{x}^{2}/n=4{\beta }^{2}{\omega }^{2}\left(1-\frac{2{\lambda }^{2}}{\pi \left(1+{\lambda }^{2}\right)}\right)/n$$

The reduction in variance associated with the estimator for $\widehat{\gamma }$ when going from the restricted model to the *partially* restricted model (with covariate $x$ included in dichotomized form) can be similarly derived.

4 $$V\left(\widehat{\gamma };{m}_{\mathrm{r}}\right) -V\left(\widehat{\gamma };{m}_{\mathrm{p}}\right)=4{\beta }^{2}{\frac{{\omega }^{2}}{\tau \left(1-\tau \right)}\left(f\left({F}^{-1}\left(\tau \right)\right)+\sqrt{\frac{2{\lambda }^{2}}{\pi \left(1+{\lambda }^{2}\right)}}\left(\tau -\Phi \left({F}^{-1}\left(\tau \right)\sqrt{1+{\lambda }^{2}}\right)\right)\right)}^{2}/n$$

We will refer to the ratio between these two variance differences as the ‘dichotomization efficiency’, $D$.

$$D=\frac{V\left(\widehat{\gamma };{m}_{\mathrm{r}}\right) - V\left(\widehat{\gamma };{m}_{\mathrm{p}}\right)}{V\left(\widehat{\gamma };{m}_{\mathrm{r}}\right) - V\left(\widehat{\gamma };{m}_{\mathrm{f}}\right)}$$

5 $${D=\frac{1}{\tau \left(1-\tau \right)}\left(f\left({F}^{-1}\left(\tau \right)\right)+\sqrt{\frac{2{\lambda }^{2}}{\pi \left(1+{\lambda }^{2}\right)}}\left(\tau -\Phi \left({F}^{-1}\left(\tau \right)\sqrt{1+{\lambda }^{2}}\right)\right)\right)}^{2}/\left(1-\frac{2{\lambda }^{2}}{\pi \left(1+{\lambda }^{2}\right)}\right)$$

Detailed derivations of the expressions in Eqns. 4 and 5 are presented in Additional file 1: Appendix 1.

## Real-world data

The prevalence and extent of skewness in real-world data was explored using publicly available summary statistics on BMI, weight and lipid measurements from the US Center of Disease Control and Prevention (CDC) [25–27]. Using the provided percentile values for the variable being summarized, we used Maximum Likelihood Estimation (MLE) to find the SN parameter values that optimize the fit. Results are presented in Table 1. BMI and Weight data are specific to people aged 20. All lipid parameter data are for people aged 20–74 between 1976 and 1980.

**Table 1:** MLE parameters for fitting a SN model to common anthropomorphic and lipid measurements

Data	Location	Scale	Shape (${\varvec{\lambda}})$
Male BMI	19.4	5.51	4.55
Female BMI	17.7	6.66	7.49
Male Weight	57.9	19	3.51
Female Weight	46.8	17.7	5.19
Total Cholesterol	163	65.7	2.19
HDL Cholesterol	32.1	17.5	2.93
non-HDL Cholesterol	116	67.7	2.2
Fasting Triglycerides	49.6	115	11

**Fig. 1:**
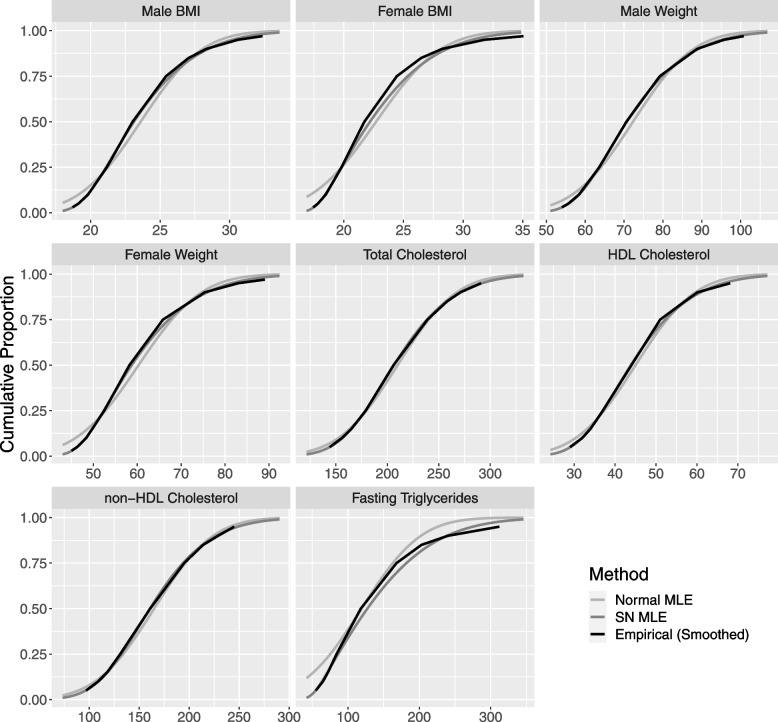
Cumulative Distribution Functions (CDF’s) for common anthropomorphic and lipid measurements with SN densities with parameters determined by MLE overlaid

The CDF of a random variable is a function with argument ‘x’ which provides the probability of obtaining a value smaller than x. In Fig. 1 it was decided to present graphs of the CDF rather than the probability density function (PDF), which is the derivative of the CDF, because the publicly available datasets provided values at non-equally spaced percentiles, which makes presentation of the PDF cumbersome and difficult to interpret. Figure 1 shows that the SN model fit the data extremely well in six of the eight cases, and moderately well in the other two (Female BMI and Fasting Triglycerides). In contrast, the normal model achieved very good fit for just two of the eight cases (Total Cholesterol and non-HDL Cholesterol). Table 1 shows that in all eight cases the amount of skewness was at least moderate ($\lambda >2$), and in three cases it was substantial ($\lambda >5$). This highlights the prevalence of skewed data in medical datasets and the importance of considering implications for analysis. In all cases the skewness was to the right $\left(\lambda >0\right)$.

## Results

**Fig. 2:**
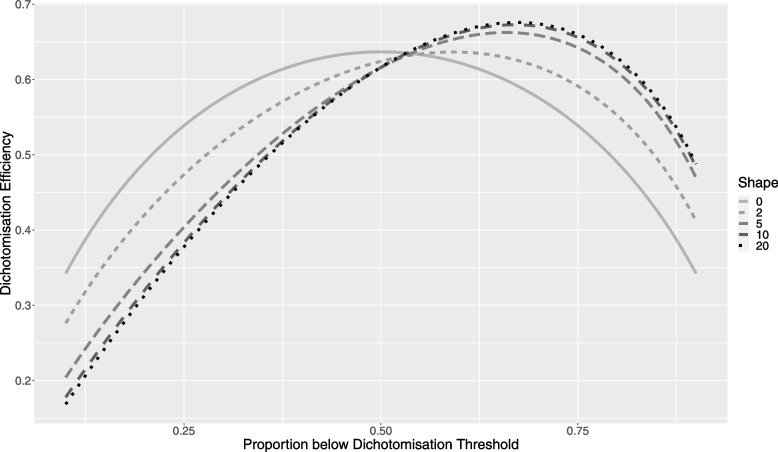
Dichotomization Efficiency as a function of Proportion below the Dichotomization Threshold for a range of shape parameters and dichotomization thresholds

**Fig. 3:**
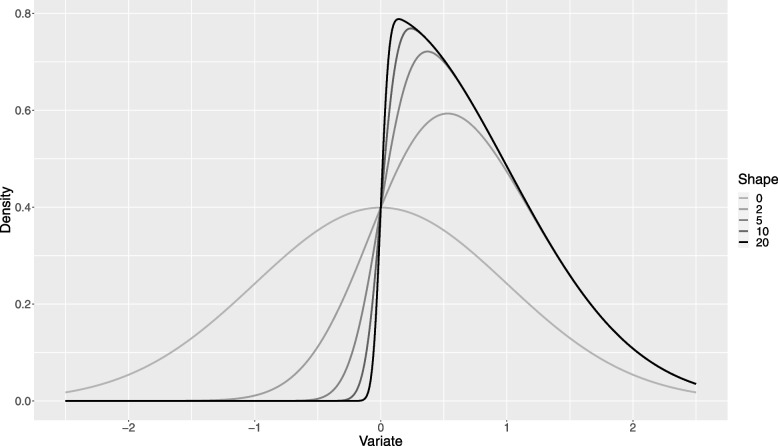
Distribution functions of standard skew-normal distributions for a range of shape parameters

We see from Eq. 5 that the dichotomization efficacy is a function of just two parameters, the SN shape parameter $\lambda$ and the dichotomization percentile $\tau$. Equation 5 may then be used to graph the dichotomization efficacy as a function of these parameters. Figure 2 shows results for a range of realistic shape parameters ($\lambda )$, with the dichotomization percentile ranging from 0.1 to 0.9. Figure 3 shows the distribution functions for the same range of shape parameter values, chosen to cover those observed in the real-world data summarized above.

Figure 2 shows that the loss of efficiency when dichotomizing a continuously valued covariate is similar for a SN distributed covariate as is the case for a normally distributed covariate ($\lambda =0)$. As for the normal case, the loss of efficiency is substantial and should be avoided if at all possible. However, if dichotomization is necessary, advice regarding the best cut-point at which to dichotomize in order to mitigate this loss should consider the likely skew in the data. For data with little or no skew, the ideal cut-point is at the median, with little additional loss so long as the cut-point remains in the percentile range 0.35, 0.65. However, when skew becomes substantial (> 5), this advice changes. The ideal cut-point becomes $\sim \frac{2}{3}$ and the acceptable range runs from ~ 0.5 to ~ 0.8. Table 2 shows the cut-point that optimises the loss of precision, as well as the range of cut-points such that the additional loss of precision is kept within modest bounds, as percentiles of the covariate being dichotomized.

**Table 2:** Optimal dichotomization cut-point, as well as minimum and maximum cut-points that avoid substantial additional loss of precision (taken as keeping the dichotomization efficacy > 0.6), as a function of the shape parameter of the SN distribution

Shape	Optimal	Minimum	Maximum
0	0.5	0.35	0.65
2	0.59	0.44	0.73
5	0.66	0.48	0.81
10	0.67	0.48	0.82
20	0.67	0.48	0.82

## Simulation

**Fig. 4:**
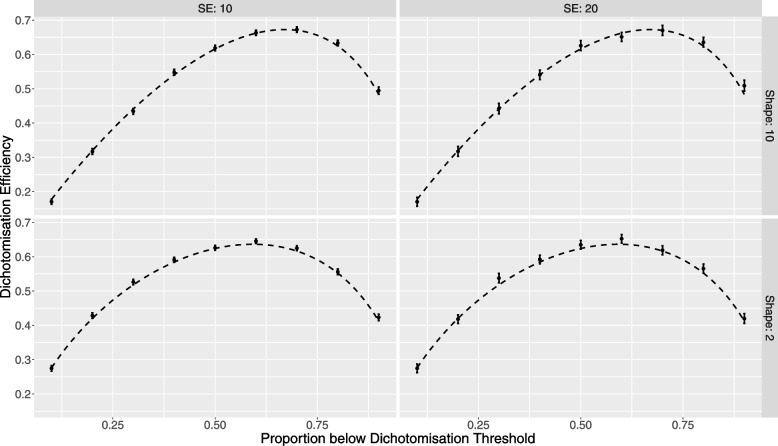
Confirmation of analytic findings by simulation, for low and high values of skewness ($\lambda =2, 10)$, and different values of the error standard deviation (10, 20)

Analytic findings were corroborated using simulation as follows. A dichotomously valued variable represented the trial arm ($z$). A continuously valued covariate ($x$), designed to have a relationship with the outcome as described below, was controlled for. The proportion below the dichotomization threshold, $\tau$, was set to values ranging from 0.1 to 0.9 in increments of 0.1, and 500 datasets were generated at each setting of $\tau$ to ensure sufficient accuracy in simulation-based estimates. The expected difference between arms ($\gamma$) was set to 15 units, and the standard error of the residuals was set to 30 units, which gives a moderate effect size of ½). The sample size per dataset was set to 100 per arm, large enough to obviate any small sample effects. The strength of the relationship between the covariate and the outcome variable was set by choosing a value of 20 for $\beta$. For each dataset at each of the above settings of $\tau$, three models were generated: the full model; the restricted model; and the partially restricted model. These were used to empirically calculate the dichotomization efficiency as a function of the proportion below the dichotomization threshold. Theoretical values based on Eq. 5 are shown in Fig. 4 (black dashed curve), and simulation-based point estimates and their 95% CI’s from the 500 runs are shown as points with error bars.

It is of note that the calculation of the dichotomization efficiency (Eq. 5) involves a division in which the denominator is a random variable. That being the case, simulation runs where the denominator has a low value due to sampling variation have high variance and thus increase the standard errors in the estimate of $D$. To circumvent this, point estimates and 95% CI’s for $D$ were calculated by regressing $V\left(\widehat{\gamma };{m}_{\mathrm{r}}\right) - V\left(\widehat{\gamma };{m}_{\mathrm{p}}\right)$ on $V\left(\widehat{\gamma };{m}_{\mathrm{r}}\right) - V\left(\widehat{\gamma };{m}_{\mathrm{f}}\right)$ and estimating $D$ from the slope of the regression line.

## Discussion

The development above is valid where the covariate to be controlled for is linearly associated with the outcome variable. Deviations from this assumption will change results. If the nature of the non-linearity is such that the dichotomisation threshold coincides with a natural ‘change point’ (i.e. a near discontinuity) in the covariate – outcome relationship, then the deleterious effects of dichotomisation may be ameliorated, or even reversed. However, such change points are rare in nature, and given that dichotomisation thresholds are not usually chosen with this in mind, such an occurrence would be purely serendipitous. Since the nature of possible non-linearities is diverse, and any attempted transformation (logarithmic, quadratic, square-root, sigmoid) will likely only partly capture it, a full investigation of their effect is considered beyond the scope of this work.

It is of note that the dichotomization efficiency for the case of a normal covariate is analogous to that demonstrated in Senn [28] for dichotomization of a normally distributed outcome variable. However, in the case of dichotomization of a covariate, the dichotomization efficiency multiplies the maximum possible gain in efficiency, which would be achieved when the covariate is left in its raw form.

Taking practical advantage of the findings in this work requires that a method to estimate the parameters of the SN distribution be available. There are a number of ways in which this can be done. One is to find the maximum likelihood estimates of the parameters using a simplex method such as that of Nelder and Mead [29]. This is the approach which was taken for estimating the parameters of the publicly available CDC datasets discussed in the Real-World Data subsection above. Alternatively, Thiuthad and Pal [30] present an approximation based on the method of moments. An R package [31] to perform this parameter estimation based on the method of Fernandez and Steel [32] is also available.

It is of interest to compare this work to a related work by Kahan and Morris [1]. Kahan and Morris consider a somewhat different but nonetheless related scenario, in which paired continuous valued data are analysed using an independent groups t-test to test for a difference between groups. They show that by ignoring the pairing when conducting the t-test, the model estimated variance of the treatment difference is inflated by a factor of ${\left(1-\uprho \right)}^{-1}$, where $\uprho$ is the correlation between the group means induced by the pairing. We can equivalently represent a paired t-test as a bivariate linear regression with treatment assignment as the predictor variable, controlling for a second categorical variable representing the participant. By assigning each participant to both of the treatment conditions, we effectively stratify by participant in the randomization, with exactly 2 observations in each stratum, one for each treatment condition. Then, by including the participant indicator in the regression at the analysis stage, this stratification variable is controlled for. Such a model is equivalent to a paired t-test, and a model which fails to control the participant indicator is equivalent to an independent groups t-test. There is a direct analogy between the relationship between the paired and independent groups t-tests, and the relationship between the full model and the restricted model in this current work. The first step in the current work – comparison of the full model to the restricted model, is exactly analogous to that of Kahan and Morris, except that in this current work the covariate to be controlled for is a continuous valued SN covariate ($x$) as opposed to being a participant indicator. The next step, which constitutes the main message of this work, is to determine what proportion of this loss in efficiency is ‘recouped’ by including the dichotomized $x$ in the model (partially restricted model) rather than its raw value.

## Conclusion

We have found that the ratio of the additional variability incorporated into the treatment effect estimate under a model with a dichotomized SN covariate to that incorporated under a model with the same covariate omitted altogether is a function only of two parameters – the proportion below the dichotomization boundary, and the shape parameter of the SN covariate, which controls the skewness. We have provided an analytic expression for this ratio which can be easily computed using any standard statistical software package. We have further shown that dichotomization of a SN covariate has a similar effect on efficiency to that of dichotomization of a normal covariate. We have also shown that in real-world medical data the amount of skewness is often substantial and that, should dichotomisation be unavoidable, this changes advice regarding the optimal dichotomization cut-point from being at the median to being at approximately the 67^th^ percentile (for right-skewed data).

## Limitations

Computation of the dichotomization efficiency depends on calculation of the cumulative distribution function of the SN distribution for which there is no closed form expression. However, it can be expressed in terms of Owen’s function [33], for which fast and accurate computational algorithms are well established [34].

The findings are asymptotically valid for large sample sizes, regardless of whether randomisation was simple, or stratified by the dichotomized covariate. For small sample sizes, findings are approximate. However, for reasonable sample sizes the magnitude of the inaccuracy is very small (of order $\frac{n-1}{n}$ with sample size $n$). Results shown in the Simulation section show that with sample sizes as small as 100 per arm, theoretical calculations match empirical findings with high accuracy.

## Software implementation details

All simulation code was written in the R programming language, version 4.1.0 [35]. Regressions used the glm function in the ‘stats’ package and all graphs were produced using the ggplot2 [36] package. Densities and cumulative densities of the skewed-normal distribution were calculated using the ‘sn’ package [37]. MLE estimation was performed using the bbmle package [38].

## Supplementary Information

**Additional file 1. **

## Data Availability

All real-world data come from the Centers for Disease Control and Prevention, are publicly available and can be found at https://www.cdc.gov/growthcharts/data/zscore/bmiagerev.xls and https://www.cdc.gov/growthcharts/data/zscore/wtage.xls. No other data were analysed in this study, aside from simulated synthetic data.
