# Supplementary material for: The effect of dichotomization of skewed adjustment covariates in the analysis of clinical trials
Source: BMC Med Res Methodol. 2023 Mar 13;23:60. doi: 10.1186/s12874-023-01878-9 (PMC10009982; doi:10.1186/s12874-023-01878-9)
Supplement: Supplementary file 1 — Additional file 1. [file 12874_2023_1878_MOESM1_ESM.docx]

**The Effect of Dichotomization of Skewed Adjustment Covariates in the Analysis of Clinical Trials**

Appendix File

Author: Alan Herschtal^[[1]](#footnote-1)*^

* Correspondences at: Alan Herschtal, [Alan.Herschtal@monash.edu](mailto:Alan.Herschtal@monash.edu)

# **Appendix 1**

Appendix 1 shows the derivation of the dichotomization efficiency, $D$. We commence by reminding ourselves of the variance of $\hat{\gamma}$ under the restricted and partially restricted models, $V\left( \hat{\gamma};m{}_{r} \right)$ and $V\left( \hat{\gamma};m{}_{p} \right)$ respectively, provided in the Methods section.

$$V\left( \hat{\gamma};m{}_{r} \right)={4\left( \sigma_{\varepsilon}^{2}+\beta^{2}\omega^{2}\left( 1-\frac{2\lambda^{2}}{\pi\left( 1+\lambda^{2} \right)} \right) \right)}/n$$

$$V\left( \hat{\gamma};m{}_{p} \right)={4\left( \sigma_{\varepsilon}^{2}+\beta^{2}\left( V\left( x \right)-V\left( x_{d} \right) \right) \right)}/n$$

where

$$V\left( x_{d} \right)={\frac{\omega^{2}}{\tau\left( 1-\tau\right)}\left( f\left( F^{-1}\left( \tau\right) \right)+\sqrt{\frac{2\lambda^{2}}{\pi\left( 1+\lambda^{2} \right)}}\left( \tau-\Phi\left( F^{-1}\left( \tau\right)\sqrt{1+\lambda^{2}} \right) \right) \right)}^{2}$$

and $V\left( x \right)=\omega^{2}\left( 1-\frac{2\lambda^{2}}{\pi\left( 1+\lambda^{2} \right)} \right)$ (Eqn. 2).

We note that both $V\left( \hat{\gamma};m{}_{r} \right)$ and $V\left( \hat{\gamma};m{}_{p} \right)$ contain a term $\frac{{4\sigma}_{\varepsilon}^{2}}{n}$, and these cancel in the subtraction.

$V\left( \hat{\gamma};m{}_{r} \right) -V\left( \hat{\gamma};m{}_{p} \right) = {4\left( \beta^{2}\omega^{2}\left( 1-\frac{2\lambda^{2}}{\pi\left( 1+\lambda^{2} \right)} \right) \right)}/n -{4\left( \beta^{2}\left( V\left( x \right)-V\left( x_{d} \right) \right) \right)}/n$

Noting that $\frac{4\beta^{2}}{n}$ is a common factor, and then replacing $V\left( x \right)$ and $V\left( x_{d} \right)$ by their full expressions in terms of the SN parameters and the dichotomisation threshold, yields

$$V\left( \hat{\gamma};m{}_{r} \right) -V\left( \hat{\gamma};m{}_{p} \right) = {4\beta^{2}\left( \omega^{2}\left( 1-\frac{2\lambda^{2}}{\pi\left( 1+\lambda^{2} \right)} \right)- \left( V\left( x \right)-V\left( x_{d} \right) \right) \right)}/n$$

$$= \frac{4\beta^{2}}{n}\left( \omega^{2}\left( 1-\frac{2\lambda^{2}}{\pi\left( 1+\lambda^{2} \right)} \right)- \left( \omega^{2}\left( 1-\frac{2\lambda^{2}}{\pi\left( 1+\lambda^{2} \right)} \right)-{\frac{\omega^{2}}{\tau\left( 1-\tau\right)}\left( f\left( F^{-1}\left( \tau\right) \right)+\sqrt{\frac{2\lambda^{2}}{\pi\left( 1+\lambda^{2} \right)}}\left( \tau-\Phi\left( F^{-1}\left( \tau\right)\sqrt{1+\lambda^{2}} \right) \right) \right)}^{2} \right) \right)$$

Then cancel $\omega^{2}\left( 1-\frac{2\lambda^{2}}{\pi\left( 1+\lambda^{2} \right)} \right)$ as a common term.

$$V\left( \hat{\gamma};m{}_{r} \right) -V\left( \hat{\gamma};m{}_{p} \right) = \frac{4\beta^{2}}{n}{\frac{\omega^{2}}{\tau\left( 1-\tau\right)}\left( f\left( F^{-1}\left( \tau\right) \right)+\sqrt{\frac{2\lambda^{2}}{\pi\left( 1+\lambda^{2} \right)}}\left( \tau-\Phi\left( F^{-1}\left( \tau\right)\sqrt{1+\lambda^{2}} \right) \right) \right)}^{2}$$

This is as presented in the Method section. We now recall that the dichotomization efficiency, $D$, is defined as $D=\frac{V\left( \hat{\gamma};m{}_{r} \right) - V\left( \hat{\gamma};m{}_{p} \right)}{V\left( \hat{\gamma};m{}_{r} \right) - V\left( \hat{\gamma};m{}_{f} \right)}$

In the Method section it was found that

$$V\left( \hat{\gamma};m{}_{r} \right)-V\left( \hat{\gamma};m{}_{f} \right)={4\beta^{2}\sigma_{x}^{2}}/{n=}{4\beta^{2}\omega^{2}\left( 1-\frac{2\lambda^{2}}{\pi\left( 1+\lambda^{2} \right)} \right)}/n$$

If we divide our expression above for $V\left( \hat{\gamma};m{}_{r} \right) -V\left( \hat{\gamma};m{}_{p} \right)$ by $V\left( \hat{\gamma};m{}_{r} \right)-V\left( \hat{\gamma};m{}_{f} \right)$, and cancel common terms in the numerator and denominator, we have

$$D=\frac{\left( f\left( F^{-1}\left( \tau\right) \right)+\sqrt{\frac{2\lambda^{2}}{\pi\left( 1+\lambda^{2} \right)}}\left( \tau-\Phi\left( F^{-1}\left( \tau\right)\sqrt{1+\lambda^{2}} \right) \right) \right)^{2}}{\tau\left( 1-\tau\right)\left( 1-\frac{2\lambda^{2}}{\pi\left( 1+\lambda^{2} \right)} \right)}$$

This is identical to the expression for $D$ in Eqn. 5.

# **Appendix 2**

By the definition of the variance of a sum of random variables:

$$V\left( x \right)=V\left( x_{d}+x_{r} \right)=V\left( x_{d} \right)+V\left( x_{r} \right)+2\times Cov(x_{d},x_{r})$$

$$V\left( x_{d}+x_{r} \right)= V\left( x_{d} \right)+V\left( x_{r} \right)+2\times\left( E\left( x_{d}x_{r} \right)-E\left( x_{d} \right)E\left( x_{r} \right) \right)$$

Intuitively, substituting all values of $x$ above the dichotomisation threshold by their mean does not change the overall mean, and likewise for all values of $x$ below the dichotomisation threshold. Therefore $E\left( x_{d} \right)=E\left( x \right)$ and thus since $E\left( x \right)=E\left( x_{d} \right)+E\left( x_{r} \right)$, we have $E\left( x_{r} \right)=0$.

$$V\left( x_{d}+x_{r} \right)= V\left( x_{d} \right)+V\left( x_{r} \right)+2\times E\left( x_{d}x_{r} \right)$$

Since $x_{d}$ can only take one of two values, $u_{-}$ and $u_{+}$, $E\left( x_{d}x_{r} \right)$ can be calculated by considering the two sub-distributions where$x<\tau$ and $x\geq\tau$. Define a random variable $x_{r-}$ as being equal to $x_{r}$ whenever $x<\tau$ and equal to 0 whenever $x\geq\tau$, and a 2^nd^ random variable $x_{r+}$ as being equal to 0 whenever $x<\tau$ and equal to $x_{r}$ whenever $x\geq\tau$. Then $E\left( x_{d}x_{r} \right)$ is the weighted sum of the means of these 2 sub-distributions.

$$E\left( x_{d}x_{r} \right)=u_{-}E\left( x_{r-} \right)\tau+u_{+}E\left( x_{r+} \right)\left( 1-\tau\right)$$

Since $x_{r-}$ is the residual value of $x$ around $u_{-}$ when $x<\tau$, $E\left( x_{r-} \right)=0$. The same applies to $x_{r+}$. Thus $E\left( x_{d}x_{r} \right)=0$. Thus $V\left( x_{d}+x_{r} \right)= V\left( x_{d} \right)+V\left( x_{r} \right)$.

1. Monash University

   School of Public Health and Preventive Medicine

   553 St Kilda Rd

   Melbourne 3004, Australia

   Alan.Herschtal@monash.edu [↑](#footnote-ref-1)
